# Iran diabetes research roadmap (IDRR) study: a preliminary study on diabetes research in the world and Iran

**DOI:** 10.1186/s40200-017-0291-9

**Published:** 2017-02-17

**Authors:** Ensieh Nasli-Esfahani, Farshad Farzadfar, Marjan Kouhnavard, Robabeh Ghodssi-Ghassemabadi, Alireza Khajavi, Maryam Peimani, Rezvan Razmandeh, Mahboobeh Vala, Gita Shafiee, Camelia Rambod, Mahnaz Sanjari, Maryam Aalaa, Maryam Ghodsi, Farideh Razi, Fatemeh Bandarian, Bagher Larijani

**Affiliations:** 1 Diabetes Research Center, Endocrinology and Metabolism Clinical Sciences Institute, Tehran University of Medical Sciences, Tehran, Iran; 2 Non-Communicable Diseases Research Center, Endocrinology and Metabolism Population Sciences Institute, Tehran University of Medical Sciences, Tehran, Iran; 3 Departments of Biostatistics, Faculty of Medical Sciences, Tarbiat Modares University, Tehran, Iran; 4 Faculty of Paramedical Sciences, Shahid Beheshti University of Medical Sciences, Tehran, Iran; 5 Department of Health Education & Promotion, School of Public Health, Tehran University of Medical Sciences, Tehran, Iran; 6 Metabolic Disorders Research Center, Endocrinology and Metabolism Molecular -Cellular Sciences Institute, Tehran University of Medical Sciences, Tehran, Iran; 7 Chronic Diseases Research Center, Endocrinology and Metabolism Population Sciences Institute, Tehran University of Medical Sciences, Tehran, Iran; 8 Endocrinology and Metabolism Research Center, Endocrinology and Metabolism Clinical Sciences Institute, Tehran University of Medical Sciences, Tehran, Iran; 9 Elderly Health Research Center, Endocrinology and Metabolism Population Sciences Institute, Tehran University of Medical Sciences, Tehran, Iran; 10 Obesity and Eating Habits Research Center, Endocrinology and Metabolism Molecular -Cellular Sciences Institute, Tehran University of Medical Sciences, Tehran, Iran

**Keywords:** Diabetes mellitus, Roadmap, Trend, Bibliometrics, Iran

## Abstract

**Backgrounds:**

Diabetes is one of the most common metabolic disorders worldwide. This study aim was to provide detail analysis of diabetes research output and its trend in Iran as well as in the world and compare them.

**Methods:**

Data was retrieved from PubMed database using a suitable search strategy and application of proper operator “AND”, “OR” and “NOT”. All English documents published from 2008 to 2012 were included. Meeting abstract, letter to the editor, guidelines, consensus and reviews were excluded. Obtained documents for Iran and world were categorized in eleven groups including diabetes management, education, paediatrics, nutrition, epidemiology, diabetes complications, stem cells, gestational diabetes mellitus (GDM), psychiatrics, genetics and prevention and were compared.

**Results:**

Total number of DM publications was 59513 for world and 648 for Iran. Trend of DM publications was increasing during the 5 years with a growth rate of 22.5% for world and 23.4% for Iran. Contribution of Iran in the world diabetes output reached 1.08 in 2012. The most and the least number of DM documents were related to complications and preventions, respectively both in Iran and the world. Three leading countries with highest proportion of RCTs (randomized clinical trial) to their total DM publications were Italy, Germany and Iran.

**Conclusion:**

The most number of diabetes research was in the field of diabetes complication, management and genetics in the world as well as in Iran. During the 5-year period, despite of the world sanctions against Iran, diabetes research trend was increasing in Iran relatively parallel to the world research and sanction had no significant effect on Iran.

## Background

Diabetes, one of the most common non-communicable diseases (NCDs), is the fourth or fifth leading cause of death in most high-income countries and is certainly one of the biggest health challenges facing the world today [1]. Diabetes prevalence has dramatically increased in many countries over the past decades. According to IDF reports, 415 million people are estimated to have diabetes in 2015 which will rise to 642 million by 2040 [2].

Amongst all regions, the Middle East and North Africa Region (MENA), where Islamic Republic of Iran is belonged to, has the highest prevalence of people with diabetes. According to the latest estimates, 35.4 million people, or 9.1% of the adult population, have diabetes in MENA and this number is expected almost double by 2035 [2, 3].

Surprisingly, Iran owns the third place in terms of the total number of adult population with diabetes across the MENA region (4,602.2 adults with diabetes (20–79) in 1000 s) [2].

Diabetes imposes a large economic burden on national health care systems worldwide and so more prevention and management efforts are required to relieve this burden [4].

Health spending on diabetes accounted for 11.6% of total health expenditure worldwide in 2015. With such a high cost burden, the disease is an obstacle to sustainable economic development [2, 5].

In Iran, also, diabetes consumes more than 8.6% of total health expenditure. Diabetic patients have four times higher rates of hospitalization [6], 2.6 times higher numbers of annual physician visits and 2.5 times greater volume of drug prescriptions than non-diabetic patients [7, 8].

Today, although there is no cure for this progressive disease, new drugs and a holistic approach to treatment have improved prognosis and quality of life, morbidity and mortality *due to diabetes complications*. On the other hand, the grand challenge of curing diabetes will only be met through increased research and so obvious that success depends not only on increased funding, but also on more rational use of research budgets and better coordination [9]. The United States has been successful in this context with the National Institute of Diabetes and Digestive and Kidney Diseases (NIDDK) overseeing, coordinating and funding the federal diabetes research effort in a most effective fashion. In Europe, also the Research Directorate of the European Commission has developed a road map for diabetes research (DIAMAP) in collaboration with Alliance for European Diabetes Research (EURADIA), a non-profit organization with the mission to promote diabetes research in Europe [10, 11].

However, there is no a similar organization or institute in Iran with aims to supervise our research, and possibly resulting in precious research funds not being invested as effectively as they might.

The current approach to research we have today in Iran, with mostly random and uncoordinated investment, lags behind the international competitiveness of Iranian research [12, 13]. In this regards, this preliminary study was conducted to obtain an overview about diabetes research in the world and Iran, before developing a roadmap for diabetes research called “Iran Diabetes Research Roadmap (IDRR) study” to recognize the *challenges,* gaps and opportunities in *diabetes research in Iran.* With such research road maps in mind, IDRR study seeks to ensure that diabetes research priorities can engage with both national plans and with larger-scale more general strategic approaches to funding and coordination.

This study is a bibliometric study aimed to analyse a body of literature and report characteristics of diabetes research output and its trend in Iran and world and compare them.

## Materials and methods

“Iranian Diabetes Research RoadMap” project has been coordinated by Iranian Diabetes Research Network (IDRN) and overseen by a Project Steering Committee (PSC) which provided scientific guidance to the project. Iranian Diabetes Research Network was first established in 2002 by Endocrinology and Metabolism Research Institute (EMRI) at Tehran University of Medical Sciences in collaboration with Ministry of Health and Medical Education with the primary goals of expanding knowledge and reforming practice in all aspects of diabetes care, facilitating the translation of evidence-based knowledge into practice through active cooperation between the network members, organizing diabetes care programs for the general populations, patients and health care providers and finally conducting research projects in the field of diabetes.

To obtain and compare scientific production of the world and Iran about diabetes during a 5-year period (2008–2012) a comprehensive search was done in PubMed database using the first search strategy.

“First Search Strategy” was used to acquire results related to diabetes in the world. In this stage, PubMed search was done by using the following MeSH Terms: “Diabetes Mellitus” [Mesh] OR “Diabetes Mellitus, Type 1” [Mesh] OR “Diabetes Mellitus, Type 2” [Mesh]. To retrieve diabetes articles in lran, another search was done on PubMed database using the following keywords: “Diabetes Mellitus” [Mesh] OR “Diabetes Mellitus, Type 1” [Mesh] OR “Diabetes Mellitus, Type 2” [Mesh] AND (Iran [MeSH] OR Iranian OR Iranians OR I.R. Iran). This search strategy was considered as the “Second Search Strategy”.

All search results were limited to the years 2008–2012 and duplicate documents, meeting abstract, letter to the editor, guidelines, consensus and reviews were excluded. Also, only English documents were eligible. All documents related to Iran were removed from the world search results.

Following the first search protocol, 73299 records were obtained. Then after excluding unrelated documents according to the title (11459) a total number of 61840 articles remained. These records were evaluated based on the abstract and texts and finally 59513 documents remained that were categorised (2327 were unrelated and excluded after second screen).

The second search strategy, for retrieving Iranian publications, resulted in 723 records. In first screening, after excluding duplications and unrelated documents based on the title, 692 records remained (31 records removed). In the second screening based on the abstract and text of articles, 44 documents were unrelated and excluded and finally 648 documents remained.

Then results of each group (world and Iran) were categorized separately in eleven categories according to their titles, abstracts or full texts by the working groups. Studies irrelevant to all eleven research areas or unrelated studies were removed. Articles on more than one area where put in two or more categories.

Obtained diabetes documents were categorized in eleven scientific areas by the steering committee as following:

- Diabetes management
- Patient education
- Pediatrics
- Nutrition
- Epidemiology
- Diabetes Complications
- Stem Cells
- Gestational diabetes mellitus (GDM)
- Psychiatrics
- Genetics
- Prevention

Each working group was supervised by an expert with comprehensive scientific knowledge in the relevant area and more broadly in diabetes research.

In addition, top five countries in diabetes research were identified by transferring the primary results of the first search strategy to RefViz software and then ranked based on the corresponding authors’ country.

Since Randomized Control Trials are of high value in comparison to other types of studies, we retrieved RCTs (randomized clinical trial) by filtering the first and second search results to “RCT” and identified the total number of RCTs both in Iran and the world over the years 2008–2012. Moreover, five leading countries in conducting RCTs in the field of diabetes were also identified using RefViz software based on the corresponding authors’ country.

To evaluate annual research growth rate and compare this rate between Iran and the world a comprehensive analysis method was required. For this purpose, numbers of published papers were transformed to proportions for further analysis. For each year of study, the proportions of annual publications of each research area were calculated by dividing the number of published papers to the total number of publications in all 11 categories during 5 years. These proportions were tested for temporal trends during 5 years by Cochran-Armitage trend test (using function prop.trend.test in R). The correlations of these quantities between Iran and the world were assessed; also we measured these correlations with one-year lag. Considering diabetic RCTs publications, the equality of proportions of all RCTs to total diabetic publications for 5 years were tested by chi-square test, also the annual proportion of RCTs to total diabetic publications were calculated for each country. Pairwise correlation was calculated between each two countries. In order to find likely clusters of countries according to the proportion of RCTs publications, we implied cluster analysis through hierarchical clustering method, Using hclustvar function in R package ClustOfVar**. All analyses were conducted using R: 2.15.2.

## Results

**Fig. 1:**
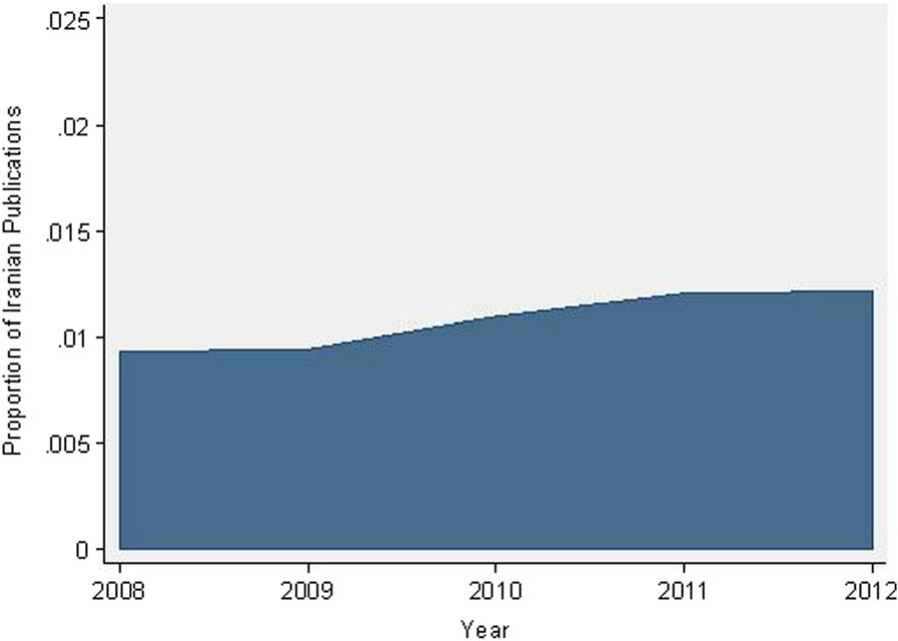
Contribution of Iranian Total DM Publications to the World Total DM Publications

During the study period (5 years), number of total DM publications was 59513 for world and 648 for Iran. Total world DM publications increased from 10519 in 2008 to 13567 in 2012 with a change rate of 22.5% during this period while contribution of Iranian publications to the world total DM publications was 0.93% in 2008 and reached 1.08% in 2012 with a change rate of 23.4% (Fig. 1).

Table 1 demonstrates the number and proportion of publications in each field of study for world and Iran. These eleven fields constitute about 70% of total worldwide DM publications and 86.1% of total Iranian DM publications. Complications and preventions had the most and the least proportions of the total DM publications during 5 years, respectively both in Iran and the world (Table 1).

**Table 1:** Field Contribution to the total DM Research of World and Iran during 5 years

	World		Iran	
Field of Study	Number of DM Publications	Percent	Number of DM Publications	Percent
Complications	13598	22.9	198	30.6
Management	6617	11.1	77	11.9
Genetics	4980	8.4	72	11.1
Epidemiology	3675	6.2	38	5.9
Pediatrics	2882	4.8	21	3.2
Nutrition	2171	3.7	53	8.2
GDM	1917	3.2	37	5.7
Education	1368	2.3	14	2.2
Stem Cells	1109	1.9	15	2.3
Psychiatrics	604	1.01	27	4.2
Prevention	470	0.79	6	0.9

**Fig. 2:**
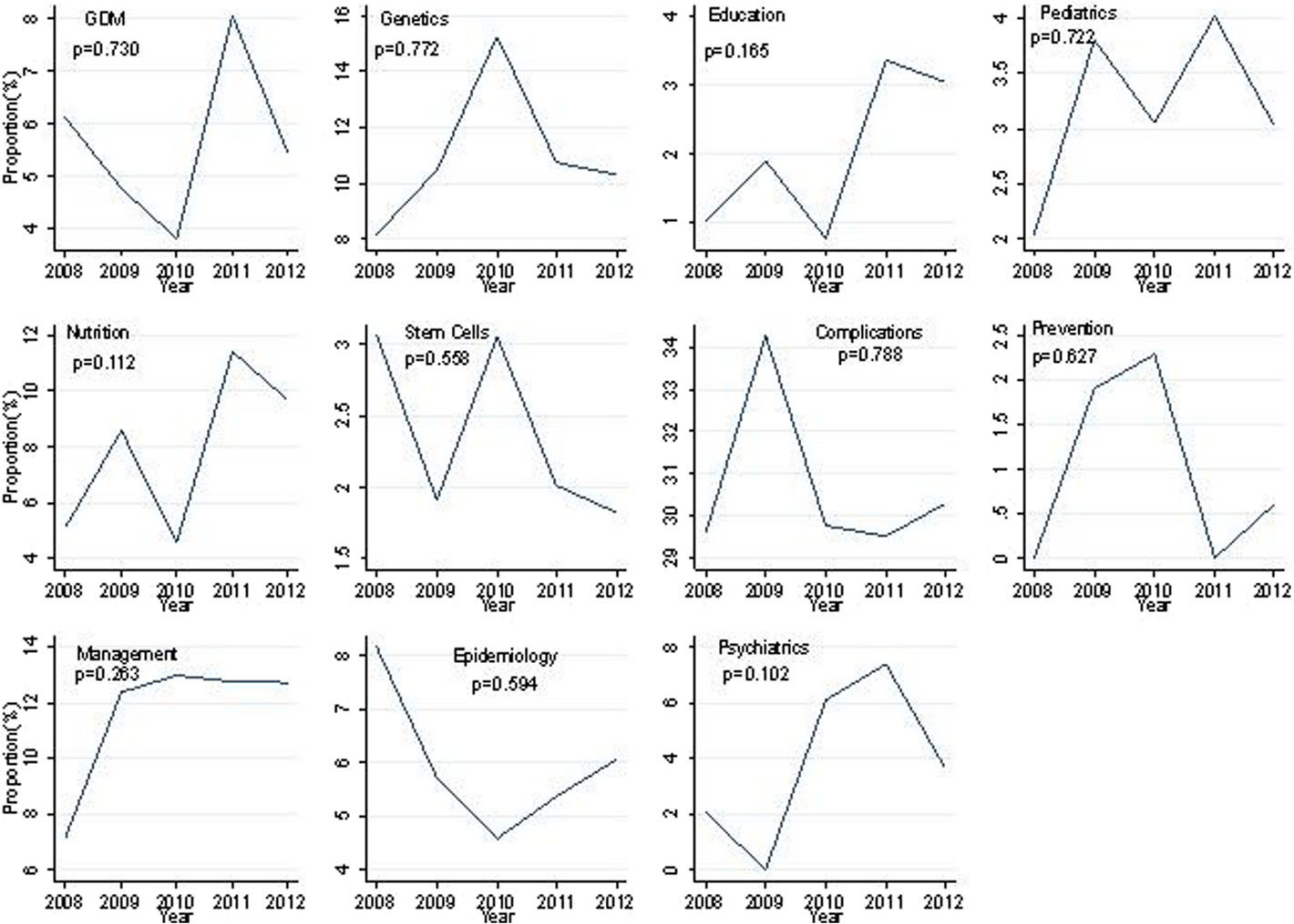
Proportion that each field accounts for in the total number of DM Publications for each year in Iran (*P*-value of trend test is represented for each field)

Figure 2 demonstrates annual trends of the number of publication of each field to the total DM publications in Iran. Complications had consistently the most yearly contribution of total DM publications. Although the annual contribution of GDM, genetics, educations, pediatrics, nutrition, management, and psychiatrics to the total Iranian publications was increasing from 2008, but unique trend was not observed in these fields.

**Fig. 3:**
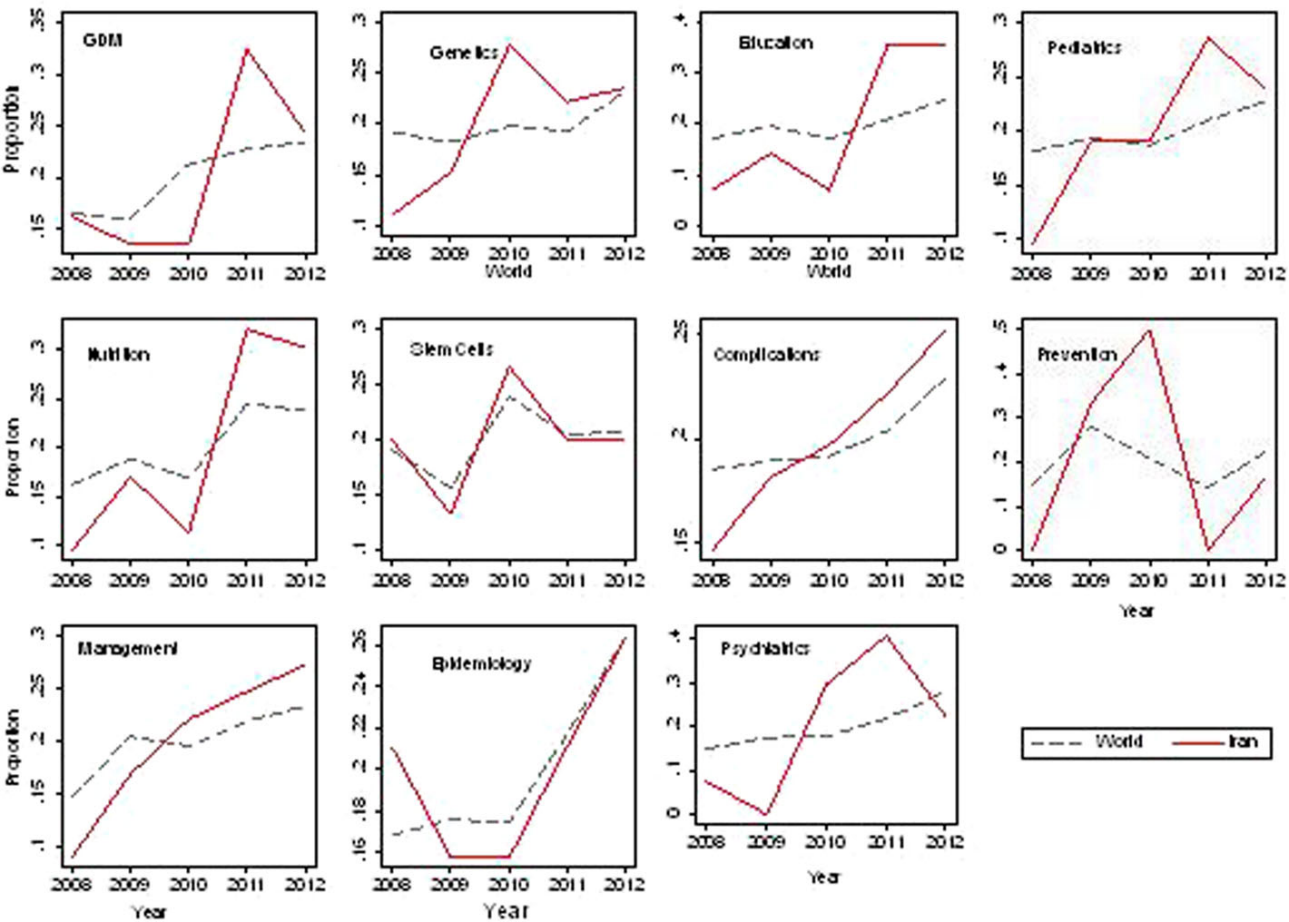
Proportion that each field accounts for in the total number of publications of each field (*dashed line*: worldwide publications, *solid line*: Iranian publications)

The impact of each field over the period of study is presented in Fig. 3. There was an increasing trend (*p* < 0.05 for trend) in the number of worldwide publications in each field of research, except prevention (*p* = 0.775 for trend) that shows fluctuation. Iran showed increasing trend with constant rates for the number of publications of complications (*p* = 0.005 for trend) and management (*p* = 0.002 for trend). Also an increasing trend was observed in the number of publications of genetics (*p* = 0.032), educations (*p* = 0.020), nutrition (*p* = 0.001), and psychiatrics (*p* = 0.004).

World RCTs constituted 11.4% of total world diabetes publications during these 5 years. Contribution of RCT publications of each country to the total number of world RCT publications are represented in Table 2. While the USA was leading among leading countries, Iran had the least contribution to the total number of RCTs (28.5% vs.1.29%). Also the proportion of RCTs of each country to its total DM publications is represented in Table 2. Italy had the most contribution of diabetic RCTs publications to its total diabetic publications (15.8%), and China had the least one (6.5%), this proportion was 15.4% for Germany. Iran was in the third place of having high contribution of RCTs to total diabetic publications (14.2%). We probed into Iran and other six leading countries’ proportion of diabetic RCTs to total Diabetic articles (Table 2).

**Table 2:** Proportion of each country RCTs to total world RCT articles and to the country total DM publications

Country	% of total country DM publications^a^	% of total world RCTs^b^
Italy	15.87	5.51
Germany	15.37	4.54
Iran	14.18	1.29
Spain	13.90	2.40
Australia	12.74	3.17
UK	12.50	6.31
USA	11.80	28.50
Canada	11.67	2.99
Japan	10.74	5.41
France	9.75	1.87
China	6.51	3.71

^a^Total DM publications of each country during years of 2008–2012

^b^Total world RCTs during years of 2008-2012

The annual proportion of world RCTs to the world total diabetes articles was 11.7, 11.6, 10.3, 11.9 and 11.3%, respectively from 2008 to 2012. There was no significant trend in proportion of annual RCT publications to the total DM publications during 2008 to 2012 (*p* = 0.757 for trend).

**Fig. 4:**
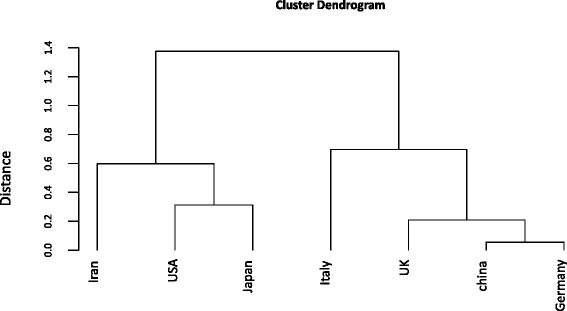
Dendrogram of Countries According to Their Proportion of Publications

From dendrogram, as a result of cluster analysis, in Fig. 4, we concluded that there were two major clusters. The first cluster consisted of the Germany, China, UK, and Italy in which Germany and China clustered together at the distance of 0.05, and then UK merged to this group at the distance of 0.23 and Italy added to this cluster at the distance of 0.7. The second cluster consists of the USA, Japan, and Iran. Japan and the USA merged together at the minimum distance of 0.3, this group differed from Iran with a distance of 0.6 and these three countries formed a new cluster. The distance between the two major clusters was 1.4.

**Fig. 5:**
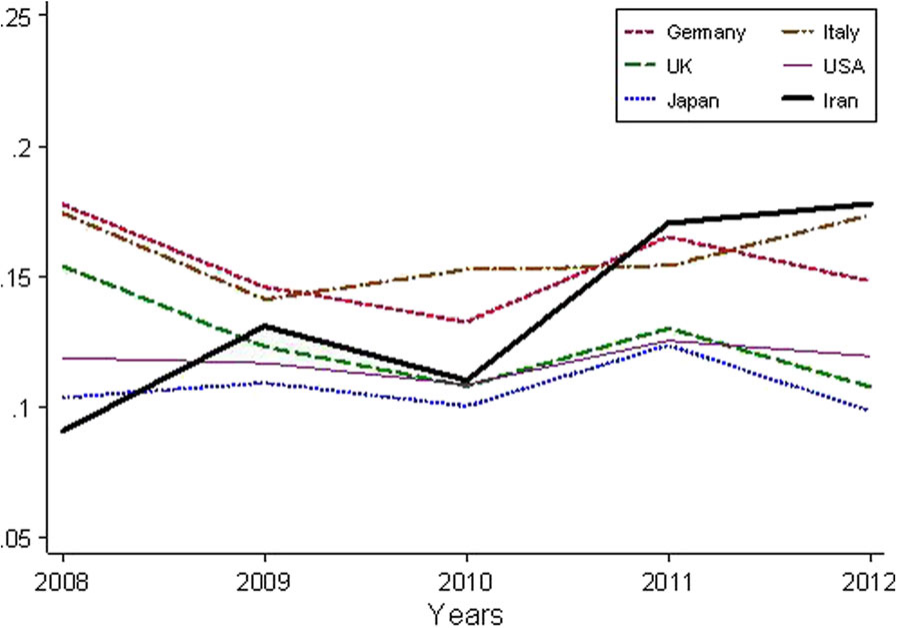
Annual proportion of RCTs to its total DM publications for leading countries

As we can see in Fig. 5, there was a uniform pattern for the proportion of diabetic RCTs to all diabetes publications for leading countries (*p* > 0.05 for trend, results are not shown), an exception observed for UK which had a decreasing pattern (*p* = 0.023 for trend) but Iran showed an increasing trend during these 5 years (*p* = 0.025 for trend).

## Discussion

This study showed increasing trend of Iran and world DM publications during the study period (2008–2012). Iranian researchers in this period published 648 articles about diabetes while at the same time 59513 articles about DM was published in the world. Proportion of RCTs to other diabetes research in Iran (14.1%) was higher than US and UK (11.8 and 12.5%, respectively). Italy and Germany had highest proportion of RCTs compared to other countries (15.87 and 15.37%, respectively).

The first bibliometric study about diabetes research output in Iran was conducted by Rezaei et al. In that study, the number of DM publications by Iranian authors increased from zero to 9 from 1992 to 2002 and Iran publications trend had the highest growth rate among Middle East countries. Contribution of Iran in the world DM scientific production in that time was only 0.2% and Iran took the fourth place after Saudi Arabia, Kuwait and Egypt among Eastern Mediterranean region countries [14].

In Rasolabadi et al., recent study about world diabetes research output, Iran cumulative DM publications until the end of 2014 was 4425 documents and annual growth rate of Iran scientific production in DM was 25.5% during the same period. However, this growth rate was negative and decreased to 14.16% between the years 2013 and 2014. These findings are similar to ours [15].

Also, in that study among the top 25 world countries, Iran with a global share of 0.7% took 25^th^ place in production of the world diabetes knowledge [15]. In our study contribution of Iran in the world diabetes knowledge production with a growth rate of 23.4% was 1.08% during 5 years of study (from 2008 to 2012) which is higher than the world global growth rate (22.5%).

According to Peykari et al study, between 1990 and 2012, three countries of Turkey (30.2%), Israel (27.4%), and Iran (12.7%), respectively were the first three leading countries in the region that produced about 70% of DM knowledge in the region. The overall growth rate of Iran publications was 9% in the same period [16]. In our study, about 10 years after the first report [14], Iran contribution in the world DM output only increased 0.88% compared to the first report and reached to 1.08% and took third place in the region [16]. However, as the duration of Rasolabadi et al study was longer than ours, global share of Iran was reported 0.7% in that study from the beginning until the end of 2014 [15]. Contribution of Iran in diabetes output production in the Middle East region was reported 12.7% by Peykari et al in a 22-year period from 1990 to 2012 [16].

Also, citation of Iran diabetes publications increased during the study period and reached the highest number in 2014. Average citation per paper in that study was reported 10.4 in 2014 which is higher than prior years [15].

Geaney et al. in their recent study evaluated world diabetes research output and found that the greatest number of documents were published in 2010 and United States had the greatest contribution in overall output (28.8%) followed by United Kingdom (8.2%) and Japan (7.58%) [17]. The same results was reported by Rasolabadi et al study in Iran [15].

Also, in another study Indian diabetes research outputs were analyzed. That study evaluated India publication trend between 1999 and 2008 and reported an annual average growth rate of 13.7% [18]. Gupta et al., classified diabetes research output under seven broad subject areas and showed that medicine, followed by biochemistry, genetics and molecular biology (28.6%), pharmacology, toxicology, and pharmaceutics (22.9%), agricultural and biological sciences (7.4%), chemistry (6%), neurosciences (2.9%) and immunology and microbiology (2.5%), respectively were the most common subject of these studies [18]. In their study, from 1999 to 2008, United States, Britain and Japan, respectively were the first three countries with the highest contribution in the world diabetes scientific output [18]. In Geaney et al study in 2012 again the same countries were three leading countries in the world scientific production about diabetes [17].

Our study showed that diabetes research field in Iran was relatively comparable to the world and the highest number of diabetes field production was related to diabetes complication followed by management and genetics. However, the ratio of Iran scientific production in the field of diabetes complications, nutrition, genetics and psychiatry to its total diabetes scientific production was higher than the same ratio of the world in the same period.

Although diabetes research in Iran is to somewhat in line of the world, but it is time to move from quantity to quality and more focus on production of high quality documents with higher level of evidence (level A and B) such as RCTs, systematic review and meta-analysis as well as evaluation of interventions, HSRs (Health System Research) and cost analysis study. More attempts should be made to increase the quality of RCTs to be used in our future diabetes guideline [13].

Future studies with a comprehensive search strategy is required to evaluate research output in different fields related to diabetes (such as management, complication, nutrition…) in Iran and compare with the world and follow their trend in the next years.

## Conclusion

In conclusion, it seems that despite of the world sanctions against Iran and limited access to the resources during the study period, diabetes research has not been affected in Iran [12] and it is growing relatively parallel to the world research with few limitations. The trend of Iranian diabetes output was increasing during the 5-year period (2008–2012) although it was not equal and constant in different subject fields of diabetes in all years. It is predicted to have more and better collaboration with the world and considerable improvement in the quality and number of diabetes research production after removing world sanctions and progression of BARJAM (Iran nuclear deal with world powers) execution after 2015.

## Abbreviation

DIAMAP: Diabetes map
DM: Diabetes Mellitus
EMRI: Endocrinology and Metabolism Research Institute
EURADIA: European Research Area in Diabetes
GDM: Gestational Diabetes Mellitus
IDRN: Iranian Diabetes Research Network
IDRR: Iran Diabetes Research Roadmap
MENA: Middle East and North Africa Region
NCD: Non-Communicable Diseases
NIDDK: National Institute of Diabetes and Digestive and Kidney Diseases
PSC: Project Steering Committee
RCT: Randomized Controlled Trial
